# Use of Low Level Laser Therapy for Oral Lichen Planus: Report of Two Cases

**Published:** 2013-12

**Authors:** O Mahdavi, N Boostani, HH Jajarm, F Falaki, A Tabesh

**Affiliations:** a Dept. of Oral and Maxillo Facial Medicine, School of Dentistry, Shahid Sadoughi University of Medical Sciences, Yazd, Iran.; b Resident, Dept. of Anesthesiology, School of Medicine, Shahid Sadoughi University of Medical Sciences, Yazd, Iran.; c Dept. of Oral Medicine, School of dentistry, Mashhad University of Medical Sciences, Mashhad, Iran.; d Resident, Dept. of Oral and Maxillo Facial Medicine, School of dentistry, Shahid Sadoughi University of Medical Sciences, Yazd, Iran.

**Keywords:** Oral lichen planus, Treatment, Low level laser therapy

## Abstract

Oral Lichen Planus is a chronic inflammatory disease of unknown etiology. Erosive/ ulcerative oral lichen planus is often a painful condition that tends to become malignant, urging appropriate therapy. Laser therapy has recently been suggested as a new treatment option without significant side effects. This article presents two cases of erosive/ ulcerative oral lichen planus, who had not received any treatment before, treated with 630 nm low level laser. Lesion type and pain was recorded before and after treatment. Severity of lesions and pain were reduced after treatment. Low Level Laser Therapy was an effective treatment with no side effects and it may be considered as an alternative therapy for erosive/ulcerative oral lichen planus.

## Introduction

Lichen planus (LP) is a mucocutaneous disease of unknown etiology. T Lymphocytes are responsible for its pathogenesis [1]. It usually occurs in middle-aged women, with a prevalence of 1 to 4%. Keratotic (white) and non-keratotic (erosive/atrophic/ulcerative) forms has been described [2]. Keratotic lesions are usually asymptomatic and need no therapy, while red lesions need treatment for pain and soreness as well as their malignant potential [3-4]. Treating red oral lichen planus (OLP) is still a problem, and several empirical treatments have been used including corticosteroids, griseofulvin, curcuminoid, sulodoxide, oxypentifylline, as well as the surgery, photochemotherapy, and laser [5]. Local corticosteroid is the main treatment with promising outcomes in remission and pain/soreness relief [5-6]. The drug should be used intermittently, due to the chronicity of OLP lesions, and systemic therapy may occasionally be necessitated. Side effects are common with this treatment and include mucosal atrophy, candidiasis, adrenal suppression, gastrointestinal upset, hypertension, and hyperglycemia [7-8]. However, some patients are still resistant to this treatment. Therefore, novel effective treatments are being introduced. Low level laser therapy (LLLT) has recently been used for treating erosive OLP with minimal side effects [9-11].

Physiologic effects of low level lasers on tissues are primary or secondary. Primary effects consist of vasodilatation, as well as enhancement of blood flow, lymph drainage, cellular metabolism, neutrophil and fibroblast activation, and pain stimulation threshold. Secondary effects include aggregation of prostaglandins (such as prostaglandin E2), immunoglobulins and lymphokines, as well as beta-endorphin and encephalin in the tissue, resulting in reduction of inflammation, immune response, and pain, respectively [12-14]. Several low level lasers have been used to treat oral lichen planus, including ultraviolet (waves of below 350 nm length), Helium-Neon (632 nm), and more recently, diode (a spectrum of red to infrared wave lengths, 600 to 1100 nm) lasers. These lasers have been used with different wave lengths, intensities, powers, durations, number of sessions, and therapeutic approaches (with or without tissue absorbent) [9-11, 15]. This article presents two cases of erosive/ulcerative OLP treated via 630 nm low level laser.

**Figure 1a:**
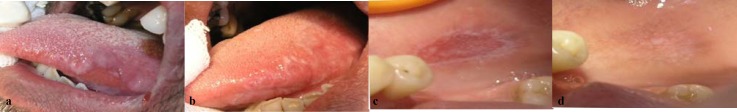
Initial tongue ulcer before treatment **b** Atrophic/keratotic lesion, e after treatment **c **Buccal erosion before treatment **d** Keratotic lesion, 1 month after treatment

## Case report

Two patients with erosive/ulcerative oral lichen planus were referred to the oral medicine department, Mashhad Faculty of Dentistry, Iran. The diagnosis was confirmed by clinical and histopathologic evaluation, with no evidence of dysplasia. No previous treatment had been given to them. They underwent laser therapy with low level red diode laser of 630 nm, 10 mill watts, 1.5 J/cm². Each lesion was emitted for 150 seconds during each session. Sessions were attended every three days during one month. The lesions were photographed each session. The patients were followed for three months, and visual analog scale (VAS) for pain was recorded before and after treatment.

**Case 1**

A 53 year-old man with severe pain due to his oral ulcer was referred to the oral medicine department, School of Dentistry, Mashhad University of Medical Sciences, Iran. A map-like ulcer, 2 cm in diameter was detected on his left tongue border with a history of two months (figure 1a). Keratotic lesions were obvious around the ulcer, as well as on the opposite border of the tongue. He was not systemically ill nor was he using medications. Other sites of his oral mucosa were intact. Marginal induration and lymph node palpation were negative. His pain was scored as 10/10 by VAS. Biopsy confirmed the clinical diagnosis of ulcerative lichen planus and no evidence of dysplasia was seen. He underwent 10 sessions of laser therapy. His pain was reduced at session three and omitted at session seven. The final lesion was atrophic/keratotic lichen planus one month after treatment (figure 1b). No significant change was recorded during next three months except for the lesion soreness in the third month.

**Case 2**

A 38 year-old woman with a 1.5-cm erosive lesion on her left buccal mucosa was referred to the oral medicine department, School of Dentistry, Mashhad University of Medical Sciences, Iran (figure 1c). The lesion appeared 3 months ago, and had a keratotic border. Keratotic papules were also present on the opposite buccal mucosa and other oral mucosal sites were intact. She had well-controlled diabetes mellitus by Glybenglamide consumption. Her initial VAS was recorded as 7/10. Histopathologic evaluation and treatment plan was similar to the case 1.The lesion changed to the keratotic type one month after treatment (figure 1d). No significant relapse was reported during the following three months of follow up.

## Discussion

Erosive/ulcerative OLP is a potentially premalignant lesion which can interfere with eating or speaking [4]. Although corticosteroids are the first line of treatment, they are not approved totally because of their side effects. Diphenhydramine or other local anesthetics might be used in conjunction with corticosteroids, as well as antifungal agents to manage Candidiasis. This multi-drug regimen reduces the patient compliance.

 LLLT is a recent evolution in medical/dental treatments, specifically regarding mucocutaneous lesions such as OLP [15-17]. Passeron et al. applied the 308 nm-excimer laser to treat erosive OLP in four patients with previous treatment failures. Twelve sessions were attended during six weeks, with the powers of 50- to 200 mJ/cm². One patient had half-part remission, two were non-responders and the other patient experienced exacerbation. The pain or soreness was not evaluated [9]. The present study showed lesion and pain remission by using a 630 nm laser, 10 sessions a month, with a power of 1.5 J/cm².

 Trehan et al. used 308-nm excimer laser in eight non-responsive painful OLP patients. The sessions were attended every week, up to 7 months. One to four hundred mJ/cm² powers were emitted. In every session, VAS was recorded and perfect photograph was taken [10]. In the study of Kollner et al., 75 to 150 mJ/cm² powers of 308 nm excimer laser were emitted to OLP lesions three times a week, up to 32 sessions. One patient showed remission after 12 sessions with no signs of relapse on a four months following period. Another patient had relative remission after 9 sessions, but with a relapse one month later. Four patients were relative and 2 patients were absolute non-responders [11]. Pain was thoroughly halted during 10 sessions of the present study, and signs of lesion evolution were absent during the three month follow up period.

 Of note, we studied lesions with no previous treatment, while others had chosen refractory cases. Also, laser powers were less in other studies compared with the present one. These facts might explain the better outcomes of the present study. On the other hand, 308-nm excimer laser emits Ultraviolet B (UV-B) rays with a tissue penetration of less than 0.3 mm; whereas 630-nm red light laser penetrates tissue several millimeters deep with proven inflammation reduction, pain relief, and ulcer healing effects [15].

 Another advantage of this study was the absence of any side effect. The UV-B excimer laser is potentially carcinogen. Besides, erythema, erosion, and soreness are other probable side effects of its use [11]. No side effect has been reported with red laser application [16-17].

 In conclusion, erosive (ulcerative) oral lichen planus can be treated by 630 nm low level laser to decrease the pain and the soreness with no side effect. Further research with more participants and statistical analyses is necessary to evaluate LLLT as a novel therapeutic approach for OLP. 
